# Clinical applications of liquid biopsy in HPV-negative and HPV-positive head and neck squamous cell carcinoma: advances and challenges

**DOI:** 10.37349/etat.2022.00099

**Published:** 2022-08-31

**Authors:** Mariana Chantre-Justino, Gilda Alves, Lucas Delmonico

**Affiliations:** 1 Research Division, National Institute of Traumatology and Orthopaedics (INTO), Rio de Janeiro 20940-070, Brazil; 2 Circulating Biomarkers Laboratory, Pathology Department, Faculty of Medical Sciences, Rio de Janeiro State University, Rio de Janeiro 20550-170, Brazil; 3 Oncoclínicas Precision Medicine, Vila Nova Conceição, São Paulo 04513-020, Brazil; University of Campania “Luigi Vanvitelli”, Italy

**Keywords:** Head and neck squamous cell carcinomas, liquid biopsy, cell-free DNA/cell-free RNA, microRNA, circulating tumor cells, extracellular vesicles

## Abstract

Head and neck squamous cell carcinomas (HNSCCs) represent the most common epithelial tumors that arise from mucosa of the oral cavity, pharynx, and larynx. The development of HNSCCs is usually associated with tobacco use, alcohol consumption, and human papillomavirus (HPV) infection. Most HNSCCs are diagnosed in advanced states, leading to a worse clinical outcome. Screening tests based on potential biomarkers associated with HNSCCs could improve this scenario. Liquid biopsy has emerged as a promising area of cancer investigation, offering a minimally invasive approach to track circulating biomarkers in body fluids that could potentially contribute to the diagnosis, predict prognosis, and monitor response to treatment. This review will discuss translational studies describing the clinical applications of liquid biopsy in HPV-negative and HPV-positive HNSCCs focused on circulating nucleic acids [cell-free DNA (cfDNA) and cell-free RNA (cfRNA)], circulating tumor cells (CTCs), and extracellular vesicles (EVs), which can be found in plasma, serum, and saliva.

## Introduction

Head and neck squamous cell carcinomas (HNSCCs) are characterized by a heterogeneous nature and are predominantly represented by malignancies that commonly arise from mucosa of the oral cavity, pharynx, and larynx [1]. In 2022, nearly 54,000 new cases of HNSCC are estimated to occur in the United States [2]. The major risk factors for HNSCC development include tobacco use, alcohol consumption, and infection by high-risk human papillomavirus (HPV), especially the HPV-16 type. HPV-associated HNSCCs are commonly represented by oropharyngeal cancers (OPCs) and account for a trend towards increased incidence rates [2, 3]. HPV-positive cases generally exhibit a more favorable prognosis compared with HPV-negative cases [1–4]. Different treatment modalities are delivered based on HNSCC characteristics. However, more advanced HNSCCs may affect treatment efficacy and lead to a challenging scenario [1].

Molecular biomarkers offering a better characterization of the diseases and early recurrences detection and/or treatment failure are desirable to improve diagnosis, prognosis, and treatment planning. Standard strategies for cancer management, like tissue biopsies and surgical interventions, are very invasive procedures with some limitations regarding the need to obtain repeated biopsies sampling to monitor the disease. Liquid biopsy is an emerging investigation in the oncology field to track potential tumor-related molecular alterations based on minimally invasive procedures through biological fluids (e.g., blood, saliva, and urine) [5]. Circulating biomarkers detected in biofluids are represented by cell-free DNA and RNA (cfDNA and cfRNA), circulating tumor cells (CTCs), and extracellular vesicles (EVs). cfDNA and cfRNA are circulating nucleic acids usually derived from processes of cell death (e.g., apoptosis, necrosis, and autophagy) or active cellularsecretions, being released into biofluids in both physiological and pathological conditions; however, differences in amounts and genetic signatures are observed between these conditions [6, 7]. CTCs are tumor cells in circulation detached from the primary tumor that are able to reach distant sites and thus facilitate the metastatic processes [8, 9]. EVs, usually represented by the exosomes subpopulation, are lipid bilayer nano-sized vesicles enriched with biomolecules (DNA, RNAs, and proteins) in their content, which allows them to participate as mediators of intercellular communication by transferring the biomolecules to regulate target cells and/or modulate the cellular microenvironment [10].

**Figure 1.**
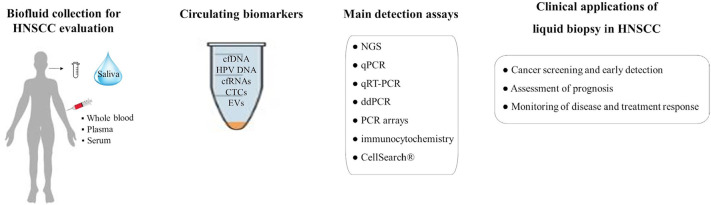
Clinical applications of liquid biopsy in HNSCC. ddPCR: droplet digital polymerase chain reaction; NGS: next-generation sequencing; qPCR: quantitative/real-time polymerase chain reaction; qRT-PCR: quantitative reverse transcription polymerase chain reaction

Since cancer is a systemic disease, the circulating biomarkers are promising molecular screening tools in clinical oncology to improve precision medicine. Regarding HNSCCs, blood (plasma/serum) and saliva are the most commonly investigated biofluids in liquid biopsy and different methodological approaches can be applied for molecular circulating biomarkers characterization (Figure 1). In this review, the relevant findings describing current clinical applications of liquid biopsy in HNSCCs were summarized, highlighting how alteration in circulating biomarkers could influence diagnosis, tumor progression, and treatment response.

## Circulating cell-free nucleic acids (cfDNA and cfRNA)

### Plasma circulating cfDNA and the diagnostic and prognostic values for HNSCC patients

#### Genetic mutations in cfDNA

The precise assessment of metastasis in the lymph node is crucial for predicting the outcome from HNSCC patients. In this scenario, plasma cfDNA may provide complementary information to clinical and imaging examinations. Schirmer et al. [11] investigated the copy number aberrations (CNAs) in plasma cfDNA from 116 HNSCC patients and 142 tumor-free control individuals by low-coverage NGS and described the copy number instability (CNI) score. CNI distinguished between patients and tumor-free individuals at a high diagnostic accuracy [area under the curve (AUC): 87.2% (95% CI, 79.4–93.3); specificity: 95.1% (95% CI, 92–97.8); sensitivity: 73.8% (95% CI, 65.9–81)], in which the presurgery CNI was increased in patients compared with controls. The CNI also increased according to tumor stage, nodal status, lymph node involvement, and tumor grade. Regarding clinical outcome, the 3-year overall survival (OS) rate for patients with CNI ≤ 72 was 90%, whereas for those with CNI > 72 was 62%. In contrast, CNI score showed no significant impact on progression-free survival (PFS). Moreover, a decrease in median CNI was found in paired pre- and postsurgery samples. Therefore, the CNI may add information on prognosis in HNSCC. A study conducted by Mes et al. [12] also evaluated plasma samples from HNSCC patients and non-cancer controls to detect CNAs by using low-coverage whole genome sequencing (lcWGS) and to detect somatic mutations by deep sequencing. By comparing tumor and plasma from 27 patients, CNAs were detected in 26 tumor biopsies (average of 34 altered segments; range 0–72) and in 52% of the corresponding plasma samples. Somatic mutations were found in 26 tumor biopsies (average of three somatic mutations; range 0–7) and in 67% of the corresponding plasma. Regarding clinical factors, the detection of CNAs and somatic mutations in plasma was significantly correlated to tumor, lymph nodes, metastasis (TNM) stage. Moreover, lcWGS was also applied for HPV DNA detection and revealed 100% of agreement between the presence of HPV DNA in plasma and HPV-positive tumors (all in oropharynx), with no detection in plasma from HPV-negative tumors or controls.

*TP53* is an important tumor suppressor gene for maintenance of cell cycle control and genome integrity. In the majority of the cancers, *TP53* is found mutated or deleted and can lead to tumor progression and metastasis [13]. A pilot study conducted by van Ginkel et al. [14] detected *TP53* mutations by ddPCR analysis in all six plasma cfDNA samples from HNSCC patients, in concordance with the *TP53* mutations previously detected by NGS in primary tumor samples. The ddPCR system is a highly sensitive technology that can efficiently detect and quantify low abundant targets like circulating tumor DNA (ctDNA), being broadly used in clinical research [15]. Khandelwal et al. [16] identified by NGS that HPV-negative patients with oropharyngeal squamous cell carcinoma (OPSCC) had a high incidence of *TP53* mutations in both plasma cfDNA and tissue samples. Additionally, the nonresponders groups to therapy exhibited mutations *TP53* R282W and *TP53* R273C, which lead to dysfunctional p53 mechanism. The R282W (c.844C>T) is a pathogenic mutation characterized by an amino acid change (arginine to tryptophan) in p53 protein that leads to a loss of hydrogen-bond interactions and disruption of the loop-sheet-helix motif. These changes impact protein thermodynamic stability and impair the DNA-binding surface, thus affecting the normal functioning of p53 [17, 18]. The R273C (c.817C>T) in p53 involves the replacement of arginine to cysteine and is also reported as a pathogenic mutation. The R273C is characterized by structural changes in the DNA-binding surface, affecting the contact with the phosphate backbone of target DNA due to the shorter lateral chains [19, 20].

Saliva specimen has been described as a suitable biological tool offering a non-invasive approach for molecular screening and clinical monitoring in HNSCC patients. Cui et al. [21] investigated single nucleotide variants in cfDNA from plasma and saliva samples of 11 patients with oral squamous cell carcinoma (OSCC) by whole-exome sequencing. The index tumors revealed *TP53* as the most frequently mutated gene (55%), followed by *CASP8*, *AJUBA*, *CDKN2A*, and *NOTCH1* mutations. The mean allele frequency of mutations in saliva was increased in patients exhibiting clinical recurrence compared with the non-recurred, being a suitable tool to determine early tumor recurrence. The authors observed that ctDNA in saliva showed greater sensitivity than ctDNA in plasma after surgery. Moreover, the concordant mutation rate between salivary cfDNA and tumor tissue DNA was 72.7%, whereas plasma cfDNA was 9.1%, suggesting that saliva samples are efficient for oral cancer surveillance. These different findings between saliva and plasma are interesting to discuss. All primary tumors in the study were collected from gingiva, tongue, or cheek, being saliva the closest contact fluid with these categories of oral tumors. The immediate release of ctDNA from such oral tumors in saliva may have concentrated the ctDNA in this fluid and contributed to a better detection of tumor-specific mutations compared with plasma samples. Since the study was conducted with a small sample size of oral cancer patients, further studies are needed.

#### cfDNA quantification

Radiochemotherapy can be applied to treat locally advanced HNSCC. However, this treatment modality can induce adverse effects, such as increased inflammatory response and toxicities [22]. The C-reactive protein (CRP) has been used as an inflammatory biomarker, but additional biomarkers are required to monitor inflammation after radiochemotherapy in HNSCC. Levels of cfDNA can be quantified in biofluids as a screening tool for early detection of cancer and to monitor relapses and treatment responses. Based on this, Zwirner et al. [23] analyzed plasma cfDNA before and during radiochemotherapy from 20 HNSCC patients, being cfDNA quantified by the Qubit fluorescence method. Pre-therapeutic cfDNA levels were associated with volumes of lymph node metastases. During radiochemotherapy, elevated cfDNA levels were significantly reported in patients treated with percutaneous endoscopic gastrostomy (PEG), treated with antibiotics, and patients with manifest infections. In addition, both cfDNA and CRP levels were significantly elevated during antibiotic treatment and after PEG placement. Furthermore, CRP values showed a significant difference between baseline and moderate inflammation, whereas cfDNA levels were significantly different between moderate inflammation and manifest infections, indicating that plasma cfDNA could be an additional biomarker and complement CRP measurements during radiochemotherapy. As also reported by the authors [23], these findings should be interpreted cautiously as confounders (infections, drugs, the tumor itself) might influence the cfDNA dynamics.

The dysfunction in mitochondrial activity may result in altered oxidative stress and influence cancer development and progression [24]. Mitochondrial DNA (mtDNA) is circular and multicopy, being the copy number of mtDNA per cell maintained in a constant range under physiological conditions, but variable according to the tumor origin. Higher mtDNA copy number has been reported in samples from untreated patients with head and neck cancers (HNCs) [25, 26] compared with samples investigated in posttreatment conditions [27, 28], suggesting that alterations in mtDNA content are important in HNCs development. Regarding circulating mtDNA, Kumar et al. [29] evaluated the levels of plasma cell-free mtDNA (cfmtDNA) and cell-free nuclear DNA (cfnDNA) from 50 HNSCC patients before treatment and 50 healthy controls by qPCR assay. As a result, plasma cfmtDNA and cfnDNA were significantly higher in cases compared with the controls and distinguished the study groups, in which cfmtDNA had a sensitivity 84%, specificity of 100% (AUC: 0.91; 95% CI, 0.692–1.0) and cfnDNA had a sensitivity of 73%, specificity of 20% (AUC: 0.73; 95% CI, 0.043–0.481). The authors also reported higher plasma cfmtDNA and cfnDNA in association with smoke and smokeless tobacco in cases than in controls, indicating that tobacco as an environmental risk factor may contribute to the HNSCC prevalence in the population of India [30].

#### Methylation signature in cfDNA

DNA methylation is an epigenetic event resulting in the reduction or inactivation of gene expression. The epigenetic inactivation by DNA methylation of tumor suppressor genes can lead to increased genomic instability, being associated with human cancer [31]. It has been reported that the two major HPV early (E) gene products, which are E6 and E7 oncoproteins, can modulate DNA methylation machinery and, therefore, DNA hypermethylation signatures are observed in HPV-related pathologies, such as cervical cancer [32], HNSCC [33], and recurrent respiratory papillomatosis [34]. By using quantitative methylation-specific PCR (Q-MSP), Misawa et al. [35] identified a methylation concordance between primary tumor samples and plasma ctDNA for ATPase sarcoplasmic/endoplasmic reticulum Ca^2+^ transporting 1 (*ATP2A1*), calmodulin-like 5 (*CALML5*), DnaJ heat shock protein family member C5 gamma (*DNAJC5G*), glycine *N*-methyltransferase (*GNMT*), glutamate-pyruvate transaminase (*GPT*), and lymphocyte antigen 6 family member D (*LY6D*) in HPV-associated OPC cases. Moreover, methylation of *CALML5* (sensitivity: 34.0%; specificity: 89.4%), *DNAJC5G* (sensitivity: 44.0%; specificity: 84.0%), and *LY6D* (sensitivity: 34.7%; specificity: 88.2%) distinguished HPV-associated OPC patients from healthy controls.

The detection of DNA methylation in *SHOX2* and *SEPT9* genes has been evaluated in plasma samples from HNSCC patients. By qPCR assays, Schröck et al. [36] initially investigated quantitative methylation levels in matched plasma and tumor tissue samples from 55 HNSCC patients and observed a significant correlation between *SHOX2* methylation in pretherapeutic plasma and methylation levels in tumor tissue, whereas a trend toward higher plasma *SEPT9* methylation was detected in cases *SEPT9* hypermethylated tumors. Plasma *SHOX2* and *SEPT9* hypermethylation were significantly higher in untreated HNSCC patients compared with non-cancer controls. Additionally, plasma *SHOX2* and *SEPT9* methylation levels were significantly associated with tumor and lymph nodes categories and histological grade, being *SEPT9* methylation an independent prognostic factor for OS, risk of recurrence, and distant metastases. Despite the encouraging results, applying the methylation assays for cfDNA in clinical routine requires attention because some healthy individuals might exhibit elevated biomarker levels (false-positives), whereas some cancer patients might show undetectable levels (false-negatives). This factor may occur due to the absence of a standard evaluation method currently recommended to describe the clinical performance of methylation biomarkers, which may influence the reproducibility of the findings. Also, the investigations on DNA methylation may involve global or gene-specific assays, qualitative or quantitative assays, which may result in different methylation rates and thus affect the clinical significance to be applied in the routine. Based on these issues, the study conducted by de Vos et al. [37] used three different algorithms to assess *SHOX2* and *SEPT9* methylation levels in plasma samples from HNSCC patients. The results revealed that the quantitative (absolute and relative quantification) and the qualitative (quasi-digital PCR) PCR-based methods showed equal performance. Additionally, both *SEPT9* and *SHOX2* biomarkers showed prognostic value for OS in all evaluation methods. Altogether, the concordant findings of the evaluation methods suggest that different platforms can be used for the investigation of both *SEPT9* and *SHOX2* methylation biomarkers for HNSCC patients.

Using ddPCR analysis, de Jesus et al. [38] evaluated plasma DNA methylation quantitation from OPSCC patients. From 20 OPSCC patients with paired tumor and plasma samples, no methylation was detected in 5 paired samples. For the remaining 15 tumor tissue with detectable methylation, plasma ctDNA methylation was found in 11/15 (73.3%) of the samples, with cyclin A1 (*CCNA1*) exhibiting higher methylation rates (7/11), followed by TIMP metallopeptidase inhibitor 3 (*TIMP3*), cadherin 8 (*CDH8*), and death-associated protein kinase 1 (*DAPK*) (2/11, 1/11, and 1/11, respectively). Post-treatment plasma collections from three cases revealed one patient with no decrease in *CCNA1* methylation levels and the other one showing increased *CCNA1* methylation. No significant association was found between DNA methylation and HPV status. Altogether, the ddPCR-based assay was feasible to detect plasma DNA methylation.

#### Circulating HPV DNA in plasma as a diagnostic and prognostic biomarker in HPV-related HNSCC

HPV-positive HNSCC cases particularly involve OPSCC patients. The screening of circulating HPV DNA [cell-free HPV DNA (cfHPV DNA)/circulating tumor HPV DNA (ctHPV DNA)] in biofluids from infected patients has been reported as a useful approach to describe the disease status and for clinical surveillance. By analyzing OPSCC patients experiencing recurrent and metastatic disease, Hanna et al. [39] showed that plasma cfHPV DNA levels evaluated by ddPCR assays correlated with tumor burden, dominant site of disease (regional or distant), and disease progression. By qPCR assays, Mazurek et al. [40] investigated OPSCC patients and observed that advanced N disease (N2 or N3) and high HPV16 DNA viral loads in tumor tissues were significant factors that resulted in higher detection of cfHPV16 DNA in plasma samples collected before treatment. Similarly, Veyer et al. [41] studied OPSCC patients at diagnosis and found a positive correlation between plasma ctHPV16 DNA values and TNM status using ddPCR detection, in which higher T and N status were significantly correlated with higher ctHPV16 DNA loads. Additionally, the authors reported that a patient with increasing ctHPV16 DNA load detected in posttreatment had recurrence and distant metastasis and died a few days later. Chera et al. [42] also used ddPCR assay and reported higher plasma ctHPV16 DNA levels at baseline in OPSCC patients with higher TNM status (T2 *vs.* T0/T1 and N2a/N2b *vs.* N0/N1) and persistent/recurrent disease for patients showing unfavorable ctHPV16 DNA clearance after treatment. Therefore, these observations are in agreement with other studies suggesting that the dynamic monitoring of circulating HPV DNA could complement imaging-based assessment for HNSCC surveillance by predicting the treatment response since patients exhibiting persistent circulating HPV16 DNA in plasma after treatment reveal residual disease and show higher risk to develop treatment failure and distant metastases [43–46].

### Circulating cfRNAs as promising biomarkers in HNSCC

RNA molecules play a crucial role in several biological processes, in which dysregulation of RNA expression is an important hallmark of cancer influencing tumor development and progression. Advances in genomic technologies have identified several types of RNA and recent studies have focused on non-coding RNAs (ncRNAs) as potential candidates for screening and monitoring of cancer [47]. The ncRNAs are abundant in the human genome and involved in different regulatory processes, being investigated to be used in specific targeted therapy [48]. The most investigated classes of ncRNAs are represented by microRNAs (miRNAs, ~22 nucleotides in length) and long non-coding RNAs (lncRNAs, > 200 nucleotides in length).

The upregulation or downregulation of circulating ncRNAs is a promising biomarker in liquid biopsy to monitor the tumor dynamics in patients with HNSCC. Rabinowits et al. [49] investigated the miRNA expression by using TaqMan-based arrays in 5 patients with tongue SCC prior to surgery and identified 9 upregulated miRNAs (hsa-miR-19a; hsa-miR-512-3p; hsa-miR-27b; hsa-miR-20a; hsa-miR-28-3p; hsa-miR-200c; hsa-miR-151-3p; hsa-miR-223; hsa-miR-20b) and 4 downregulated miRNAs (hsa-miR-370; hsa-miR-139-5p; hsa-miR-let-7e; hsa-miR30c) that were expressed in both tumor and plasma. Molecular biomarkers are desirable for the early detection of diseases and some studies have reported that circulating miRNAs can distinguish between healthy individuals and HNSCC patients. Wei et al. [50] study compared plasma miR-21 levels between 76 patients with laryngeal squamous cell carcinoma (LSCC), 40 patients with premalignant laryngeal lesions (PLLs), and 19 controls without dysplasia using ddPCR and qRT-PCR assays. The authors detected high expression of plasma miR-21 in PLLs and LSCC samples compared with the control group, describing a positive correlation of miR-21 levels between tissue and plasma samples. By NGS analysis, Pedersen et al. [51] reported that a combination of miR-30a-5p and miR-769-5p in plasma better discriminated between the 55 OSCC patients and the 18 healthy controls investigated in the study (AUC miR-30a-5p: 0.97; AUC miR-769-5p: 0.94). By comparing serum samples from 15 OSCC patients and 15 healthy controls, Bolandparva et al. [52] found higher miR-424-5p expression (1.96 times) and lower miR-138 expression (3.05 times) in samples from patients using qPCR assay. Grzelczyk et al. [53] evaluated by microarray the serum miRNA signatures from 66 LSCC patients and 100 healthy controls and observed that miR-31, miR-33, and Let-7a expression strongly differentiated these groups.

#### miRNAs expression as a biomarker for prognosis and treatment response

The clinical implications of miRNAs expression for the prognosis and treatment response have been described in HNSCC studies. Summerer et al. [54] evaluated plasma miRNAs via qRT-PCR assays and detected eight miRNAs differentially expressed (miR-21-5p, miR-28-3p, miR-142-3p, miR-186-5p, miR-191-5p, miR-197-3p, miR-425-5p, and miR-590-5p) that significantly differentiated healthy donors and HNSCC patients, in which miR-186-5p showed high sensitivity (0.938) and specificity (0.917). In addition, the circulating miR-142-3p, miR-186-5p, miR-195-5p, miR-374b-5p, and miR-574-3p showed a correlation with worse prognosis and no significant correlation was found between the circulating miRNAs and HPV status. By investigating plasma miR-155 via qRT-PCR from 280 LSCC patients, Wang et al. [55] detected higher plasma miR-155 levels in cases from stages II, III, and IV compared with early stage I, also showing a significant reduction of plasma miR-155 levels after surgery. A study conducted by Avilés-Jurado et al. [56] used the Taqman assay to evaluate serum miRNAs from 54 HNSCC patients with untreated advanced tumors (stages III–IV), showing that better disease-free survival (DFS) rates were associated with higher levels of miR-26b and lower levels of miR-155. Hou et al. [57] investigated paired plasma samples from 9 HNSCC patients in pre- and post-operative (6 months) conditions and identified downregulation of miR-21 and miR-223 and upregulation of miR-99a after surgery using qRT-PCR. The authors pointed out that these observations could reflect treatment efficacy since the miRNA levels in post-operative plasma were different from those initially found in tumor tissues. Gao et al. [58] evaluated serum miRNAs from 236 LSCC patients using qRT-PCR and identified lower miR-21 levels and higher miR-10a levels in patients with T I/II stage, N0 stage, and M0 stage, which differed significantly from the levels found in patients with more advanced TNM stages. In addition, a lower 5-year survival rate was observed with a higher ratio of miR-21 and miR-10a. Quintanilha et al. [59] investigated plasma miRNAs profile from 12 HNSCC patients by NGS to evaluate the risk of cisplatin-induced nephrotoxicity. The authors identified that miR-3168, miR-6125, and miR-4718 were upregulated and the most differentially expressed miRNAs in the nephrotoxicity group, being miR-3168 and miR-4718 expression significantly associated with a higher risk of nephrotoxicity.

#### Salivary miRNAs identified in HNSCC

Regarding salivary miRNAs analysis, Liu et al. [60] showed high miR-31 levels in saliva supernatant from OSCC patients, oral verrucous leukoplakia patients, and healthy individuals by qRT-PCR. Moreover, the authors also reported a significant decrease in salivary miR-31 after tumor resection. Interestingly, higher miR-31 levels were detected in saliva compared with plasma from OSCC patients, which might suggest saliva is a suitable biological tool for oral malignancy investigations. Romani et al. [61] investigated miRNAs in saliva supernatant by microarray and qRT-PCR, identifying 25 salivary miRNAs differentially expressed between OSCC patients and healthy controls, in which 20 miRNAs were significantly upregulated in patient samples. High miR-423-5p expression was a predictor of poor DFS and a significant decrease was observed after tumor resection, suggesting miR-423-5p is a potential prognostic biomarker for OSCC.

In order to obtain heterogeneous miRNA populations and high-resolution miRNA signatures, Salazar et al. [62] opted to conduct the miRNA analyses in whole saliva samples instead of cell-free salivary supernatants. For this purpose, miRNA PCR array and qRT-PCR were applied and the investigations showed that salivary miR-9, miR-134, and miR-191 were differentially expressed between HNSCC patients and healthy controls. Additionally, miRNA expression discriminated HNSCC patients according to anatomical sites, in which miR-222, miR-127, and miR-191 differentiated tumors in the oral cavity and pharynx; miR-9 distinguished tumors in the oropharynx and larynx; miR-191 differentiated tumors in oropharynx and pharynx; and miR-127 expression distinguished tumors in larynx and pharynx. Fadhil et al. [63] reported downregulation of miR-let-7a-5p and miR-3928 in the whole saliva from HNSCC patients compared with healthy controls via NGS and qRT-PCR. The expression levels of salivary miR-let-7a-5p and miR-3928 were significantly correlated with lymph node metastasis and tumor stage of HNSCC, suggesting these two salivary miRNAs are good biomarkers for early detection and prognosis. A study conducted by Salazar-Ruales et al. [64] also investigated miRNAs collected from the whole saliva as potential biomarkers for HNSCC diagnosis and prognosis. Using PCR arrays, the expression of miR-122-5p, miR-92a-3p, miR-124-3p, and miR-146a-5p significantly discriminated between cases and controls, with AUC of 0.73, 0.70, 0.71, and 0.66, respectively. The authors also reported differentiated expression of miRNAs by comparing HNSCC localization: miR-122-5p (larynx and oropharynx), miR-124-3p (larynx and pharynx), and miR-146a-5p (larynx, oropharynx, pharynx, and oral cavity). Salivary miRNAs expression also distinguished between HPV^+^ and HPV^–^ cases (miR-205-5p, miR-122-5p, miR-124-3p, and miR-146a-5p) and TNM stages (miR-122-5p for TNM 2 and 3; miR-124-3p for TNM 2, 3, and 4; miR-146a-5p for TNM 2 and 3; miR-92a-3p for patients on TNM3). Cinpolat et al. [65] investigated miRNAs by qRT-PCR analysis in both serum and whole saliva samples from patients with salivary gland tumors (benign and malignant tumors) and healthy controls. Although differences in serum miRNAs expression have been found between studied groups, no significant difference was identified in salivary miRNAs between benign, malignant, and control groups.

#### LncRNAs involved in HNSCC

The lncRNAs represent other classes of ncRNAs identified as potential biomarkers for clinical evaluation in HNSCC. In a study involving 100 HNSCC patients and 100 cancer-free controls, Yao et al. [66] identified three plasma lncRNAs (HOXA11-AS, LINC00964, and MALAT1) using microarray and NGS that significantly upregulated in patients compared with controls. By qPCR assay, Zhang et al. [67] detected CASC15 upregulation in plasma from OSCC patients (stages I and II) compared with oral ulcer patients and healthy subjects. By comparing plasma lncRNAs between 41 HNC patients and 39 healthy individuals via semiquantitative PCR, Fayda et al. [68] observed significantly lower plasma levels of lincRNA-p21 and GAS5 in patients. In addition, the authors identified that post-treatment plasma GAS5 levels were significantly higher in patients with partial response/progressive disease compared to those with complete response, suggesting GAS5 is an attractive biomarker in predicting treatment response for HNC patients. Łasińska et al. [69] observed 34 upregulated lncRNAs in plasma from HNSCC patients compared to healthy volunteers by the qRT-PCR method. The authors also identified changes in plasma lncRNA expression according to tumor sites. Additionally, the down-regulation of Alpha 250 was observed in patients with longer PFS and longer OS, suggesting Alpha 250 as an important prognostic biomarker for HNSCC patients.

## CTCs in HNSCC

### Detection assays and markers used for CTCs characterization

CTCs are cells that detach from the primary tumor site to distant regions of the body through the circulatory system, blood, or lymphatic pathways. CTCs are rare and range from 1–100 cells per mL of blood, being this variation dependent on tumor staging and histological subtype, in addition to the response to treatment [8]. The first description of CTCs was in 1869 by Ashworth [70], where some cells present in the blood were described as similar to those of the primary tumor. Despite their primary origin, CTCs have characteristics that facilitate the metastatic mechanism, such as mesenchymal epithelial transition properties and immune evasion [71].

Due to the rare representation in the circulation, other methods are adopted for CTCs enrichment and validation. The CTCs identification is traditionally based on epithelial markers, with epithelial cell adhesion molecule (EPCAM) being the universal marker; however, its expression is diverse in different tumors and their subtypes [71]. The presence of CTCs in the bone marrow (BM) from HNSCC patients was revealed in 1995 with the labeling of the anti-cytokeratin monoclonal antibody (A45-B/B3) directed to the cytokeratins (CKs) 8, 18, and 19 [72]. Using the immunocytochemical technique, BM aspirates from 31 HNSCC patients were evaluated and 10 of them were positive for CTCs. Most of these cases had less than 10 CTCs in one million mononuclear cells analyzed, except for one case, which had more than 200 CTCs. A detail related to the arrangement of CTCs is their distribution. In most of the analyzed cases, the CTCs were arranged in single dispersed tumor cells, while the conformation in clusters was detected in only two cases [72].

Later, in 1999, with the molecular biology techniques expansion, Brakenhoff et al. [73] used a sequence responsible for encoding the E48 antibody to reveal the presence of CTCs in the bloodstream and BM from HNSCC patients through an RT-PCR assay. Dilution points corresponding to 10, 1, and 0.1 CTCs per 7 mL of blood were performed. The authors showed signal negativity in 29 blood and BM samples from controls (non-cancer group) and patients with stages II and III HNSCC (2 and 4 patients, respectively). On the other hand, they found signal positivity in 4 stage IV cases and in 4/6 that had a recurrence. The same relationship in which CTCs are present in a higher percentage in more cancer advanced stages was observed by Wirtschafter et al. [74]. The researchers showed 8/18 (44%) cases with CTC-positive HNSCC in the bloodstream, 5 were stage IV.

The first study using the CellSearch^®^ technique (Menarini Silicon Biosystems) to detect CTCs in HNSCC patients was published in 2012 [75]. The system consists of CTCs immunomagnetic enrichment by traditional epithelial biomarkers such as EPCAM, CK8, 18, and 19, coupled to a flow cytometry (FCM) system to detect cell fluorescence by imaging. Although with only 15 cases evaluated, the system revealed 6 of them (40%) positive for CTCs, with about 2 CTCs per 7.5 mL of evaluated blood.

A recent study has demonstrated the so-called circulating hybrid cells (CHCs) in oral cavity squamous cell carcinoma [76], which consist of lineages that mix the profile of neoplastic cells with macrophages, expressing EPCAM and/or CKs, in addition to leukocyte-determining proteins, such as CD45. Interestingly, high levels of CHCs were correlated with the occult nodal metastases presence, but in contradiction, they were not correlated with the tumor stage [76]. Therefore, CTCs in HNSCC follow a traditional expression pattern of epithelial biomarkers, such as CKs. However, it is already demonstrated that in cases after tumor resection, these cells can vary from epithelial phenotype to mesenchymal, stem cells, or even, present merged profiles [77].

### CTCs and the predictive and prognostic value in HNSCC

Based on *CK19* mRNA expression to detect CTCs in the peripheral blood from 5/73 (7%) HNSCC cases, Pajonk et al. [78] have shown the correlation of these cases with the decrease in hemoglobin in the blood (anemia). Furthermore, 3 (60%) of these cases did not respond to radiotherapy and 2 of them had long-distance metastasis. The most representative data was the decrease in the mean survival, with 376 days and 703 days for those *CK19*-positive and *CK19*-negative cells, respectively. In another study, for the 6 cases that progressed to lung cancer, all presented positive expression (RT-PCR assay) for the E48 marker at the preoperative moment and consequently revealing a significant relationship for survival assessments at a median follow-up period of 36 months [79]. Likewise, CTCs were associated with the metastatic nodule’s presence in the lung, in addition to the progression and recurrence possibility of the disease [75].

Furthermore, the increased number of CTCs in the bloodstream from 53 HNC patients (majority with HNSCC) with recurrent or metastatic disease was shown to be a strong prognosis’ predictor, but low predictive value to treatment response [80]. Regarding prognosis, DFS in cases with 0, 1, and ≥ 2 CTCs was 5, 5, and 1 month, respectively; the OS for cases with 0, 1, and ≥ 2 CTCs was 9, 7, and 2 months, respectively. Regarding the predictive value, the evaluation of 10 positive cases for CTCs before and after treatment showed that five cases had an increase, one was stable and four had a decrease after treatment, revealing that CTC levels could not be correlated with primary treatment response due to non-correlation with primary tumor response. However, these data must be analyzed with caution, as there are factors intrinsic to each case, such as the presence of different subpopulations of CTCs that may be involved in survival and resistance to treatment [77]. In agreement with this statement, the CTCs detection in the bloodstream at different treatment points revealed the real prognostic value in patients with HNSCC. Inhestern et al. [81] collected 7.5 mL of blood from 40 OSCC patients with locally advanced primary tumors (stages III and IV) in the oral cavity and oropharynx (15 HPV-positive) for CTC counts before chemotherapy, before second and third chemotherapy, after surgery, before radiotherapy after surgery, and finally, at the treatment end. The data revealed that only eight (20%) of the patients did not have CTCs at the first collection point, with an average of 3,295 CTCs/mL for the others at baseline. Interestingly, there was an increase in the CTCs number after surgery and before the first radiotherapy treatment, with a mean maximum CTC during treatment of 5,005 CTCs/mL. Regarding prognosis, those patients with above-average CTCs at the first collection point had the lowest risk of DFS, and yet, patients with the highest CTCs in the overall mean post-complete treatment had the lowest OS. In this same study model, Wang et al. [82] showed an overview of blood CTC levels before and after concurrent chemotherapy and radiotherapy treatment. Blood from 53 HNSCC patients (85% in stages II-IVb) was collected 7 days before the start of treatment and 2 and 4 weeks later. The test results reinforced the greater CTCs number in advanced cases, being stage II/III, IVa, and IVb with 59.6 ± 51.4 (95% CI, 16.6–102.6), 42.6 ± 29.4 (95% CI, 32.5–52.7), and 117.6 ± 50.3 (95% CI, 81.6–153.5), respectively. Regarding the CTCs number during treatment, reduced levels were observed in all cases evaluated. However, the comparison between before and after was not significant, not even in non-responsive (stable) chemoradiotherapy. Furthermore, in relation to PFS, a significant relationship was observed between groups, 38.6 and 15.9 months for cases with decline and non-decline of CTCs after treatment, respectively. For OS, the months ranged from 41.5 to 17.7 for cases with a decline and no decline in CTC levels after treatment. In patients with locally advanced OPC (LAOC), the predictive assessment of treatment response and prognosis based on the presence and count of CTCs did not prove to be significant in the neoadjuvant chemotherapy setting. CTCs were identified in 4 and 2 (total of 21 cases evaluated) responders and non-responders’ cases (respectively) to neoadjuvant chemotherapy. Interestingly, after 21 days post-treatment, 3 responders (two of these being the same as those who had baseline CTCs) and 2 non-responders had detectable CTCs (one being the same as those who had baseline CTCs). There was no significant difference in the OS and PFS between patients with or without CTCs at baseline (*P* = 0.44 and 0.78, respectively) or day 21 (*P* = 0.88 and 0.5, respectively) [83]. Furthermore, the prognostic value of CTCs in more aggressive settings, such as in recurrent or metastatic HNSCC (r/mHNSCC) is controversial and further studies are needed. The comparison of isolation and detection methods, FCM, CellSearch^®^ (Menarini Silicon Biosystems), and EPISPOT [identification of CTCs from fluorescent labeling of *CK19*/epidermal growth factor receptor (EGFR) and estimated glomerular filtration rate], showed that the median PFS in patients with CTCs^CK19+EGFR^ (5/65) detected with the EPISPOT method on day zero (D_0_) was slightly shorter than that in patients without CTCs (3.8 months, 95% CI, 0.6–5.9, *vs.* 5.5 months, 95% CI, 4.1–6.4; *P* = 0.0518), and the CTC enumeration was not significantly associated with PFS on day twenty one (D_21_, EPISPOT, *P* = 0.1827; CellSearch^®^, *P* = 0.3304; FCM, *P* = 0.0980) [84].

The study conducted by Morgan et al. [85], using the surface-enhanced Raman scattering (SERS) technique, showed for the first time the possibility of having a cut-off point of CTCs present in the blood capable of predicting distant metastasis-free survival (DMFS) in patients with HNSCC. Of 82 cases with HNSCC, 13 experienced metastatic progressions, of which 9 had CTCs > 500/7.5 mL in blood. Statistical evaluations showed a cut-off point for DMFS of 675/7.5 mL CTCs, with 89% of patients who had ≤ 675/7.5 mL CTCs having five-year DMFS.

### HPV and CTCs

The HPV relationship (especially HPV16 and HPV18) in the progression and aggressiveness of head and neck tumors has already been elucidated [86]. However, few studies have evaluated the relationship between CTC levels and HPV-positive HNSCC tumors. Harris et al. [87] found no significant association between the presence of ≥ 1 CTC in the blood of HNSCC patients and clinical parameters (TNM stage, disease burden, HPV-positive disease, PFS, and OS). Economopoulou et al. [88] described the first study showing the presence of HPV infection in CTCs by evaluating 22 OPSCC patients with early and locally advanced disease. CTCs were isolated before and after chemoradiotherapy treatment to evaluate HPV *E6/E7*^+^ expression. Immunohistochemistry (IHC) was applied to determine cyclin-dependent kinase inhibitor 2A (p16) expression in tumor biopsies. Interestingly, the study revealed that tumor samples showing p16 positivity and HPV16 infection accompanied HPV detection in blood CTCs. For example, HPV16 *E6/E7* expression on CTCs was detected in 3/10 HPV16^+^ patients before treatment and in 4/9 HPV16^+^ patients at the end of treatment. Only two cases had HPV16 *E6/E7*^+^ before and after treatment. As expected, no HPV16-negative cases showed positive CTCs for HPV16 *E6/E7* mRNA expression. Furthermore, no significant correlation was found with *E6/E7* expression in CTCs at the end of treatment with PFS or OS. In this same context, Chang et al. [89] investigated the p16 expression in CTCs from 41 cases with HNSCC (39% in stage IV). By FCM analysis, the detection rate for p16-positive CTCs was 51.2%. The p16 positivity in tumor tissue revealed by IHC staining was not coincident with that in p16-positive CTCs evidenced by FCM. On the other hand, this correlation was found when both p16 expression and HPV genotyping were applied to tumor tissue. This difference was observed since some patients with p16-positive CTCs had tissue p16-negative IHC but HPV-positive genotyping. Regarding the prognostic profile, patients who had CTCs ≥ 3 cells/mL in blood had lower PFS. In contrast, patients with p16-positive CTCs exhibited higher PFS. Furthermore, univariate and multivariate analyses revealed that only p16-positive CTCs were found to be a prognostic factor for cancer death.

## EVs

EVs are lipid bilayer nano-sized vesicles present in physiological and pathological processes acting as mediators of intercellular communication [10]. The major EVs subtypes include microvesicles, exosomes, and apoptotic bodies, which differ especially according to their biogenesis and size [90]. Another EVs population is represented by large oncosomes, which are larger EVs (1–10 μm in diameter) and specifically released by cancer cells [91]. Since the molecular biomarkers (DNA, RNAs, and proteins) carried in EVs content, or cargo, are able to modulate the cellular microenvironment, EVs have been investigated for clinical purposes, being exosomes (30–150 nm), the most investigated EVs subpopulations. Additionally, HPV DNA/RNA can also be detected in EVs from OPSCC patients [92].

### Exosome levels in HNSCC

Exosome levels can be monitored during oncological therapy to evaluate the treatment response. Changes in exosome levels from plasma were observed in HNSCC patients during therapy (cetuximab, ipilimumab, and intensity-modulated radiotherapy), in which increased levels were found in patients who experienced a recurrence, suggesting active disease, while decreased levels were observed for those who remained disease free, suggesting therapy response [93]. In another study, a significant decrease in plasma-derived exosomes from HNSCC patients was observed during the disease-free follow-up and an increase in recurrence [94]. Since CD44v3 is reported in HNSCC progression [95], exosomal CD44v3 was also evaluated, being observed a significant decrease of CD44v3^+^/CD3^+^ after surgery and a higher ratio for non-responders compared to responders. As reported in a previous study, exosomal CD44v3 was higher in HNSCC patients compared with healthy donors [96]. Regarding immune checkpoints, exosomal programmed death-ligand 1 (PD-L1) values were significantly reduced at recurrence compared with patients who had no recurrence. PD-L1 inhibits T-cells activation thus favoring an immunosuppressive microenvironment, in which PD-L1 expression is usually found at high levels in tumor cells [97]. Therefore, cancer patients identified in this profile may benefit from immunotherapy with pembrolizumab, a PD-L1 inhibitor [98]. In a previous study, Theodoraki et al. [99] observed that patients with active disease exhibited significantly higher PD-L1^+^ exosomes than those with no evidence of disease after therapy. In addition, elevated PD-L1^+^ exosome levels were also found in patients with positive lymph nodes and higher tumor stages (III/IV). According to the authors, an explanation for the reduced exosomal PD-L1 at recurrence may be the reduced immune infiltrates observed in recurrent tumors. The authors also described those inconsistent findings are reported for HNSCC prognostic significance with tumors showing high PD-L1 expression solely by IHC findings, suggesting PD-L1 exosome-based evaluation as an alternative and attractive solution.

### EVs molecular cargo in HNSCC

The miRNAs profiling carried by EVs has also been investigated in HNSCC patients. Momen-Heravi and Bala [100] identified circulating EVs from plasma of OSCC patients enriched with miR-21, miR-27b, and miR-27a. Panvongsa et al. [101] observed that four miRNAs in plasma-derived EVs (miR-27b-3p, miR-491-5p, miR-1910-5p, and miR-630) were significantly dysregulated in locally advanced HNSCC compared with healthy donors, being ΔmiR-491-5p associated with OS and DFS. Salivary exosomal miRNAs have been investigated in OSCC patients, being found significant increase in miR-24-3p, miR-512-3p, and miR-412-3p levels in patients compared to controls [102, 103]. EV-associated proteins from plasma were evaluated and revealed 43 candidate proteins with altered abundance by comparing non-cancer controls, and oral tongue squamous cell carcinoma with and without lymph node involvement [104]. Regarding saliva, Fontana et al. [105] analyzed protein cargo from salivary small EVs of OSCC patients and identified differentially modulated proteins between groups. The results showed that 235 proteins were differentially modulated between OSCC-free and OSCC without lymph node metastasis (OSCC_NLNM); 157 proteins between OSCC-free and OSCC with lymph node metastasis (OSCC_LNM); 189 proteins between OSCC_NLNM and OSCC_LNM, suggesting that protein cargo from salivary EVs exhibits functional signature according to clinical conditions.

## Challenges and perspectives

Liquid biopsy is a promising investigation area in the oncology field, being successfully described in translational studies [11, 36, 55, 82, 101]. Besides being a minimally invasive procedure, the major clinical interest in liquid biopsy is that molecular alterations detected in circulating biomarkers may reflect the alterations found in tumor tissues, thus reducing the number of invasive biopsies to monitor the tumor process. This is particularly promising to improve HNSCC investigations. NGS and ddPCR techniques are able to efficiently detect the low abundance of cancer-related biomarkers in body fluid with higher sensitivity and specificity [106]. The diagnostic accuracy may also be assessed by other available techniques with variable cost-effectiveness, being the sensitivity/specificity parameters described with variability. A meta-analysis conducted by Zhu et al. [107] reported the diagnostic value of various liquid biopsy methods in detecting pancreatic cancer, in which variable sensitivity and specificity were observed in the 7 ctDNA studies, all PCR-based assays (sensitivity range: 27.3–97.4%; specificity range: 75.0–100.0%). Lyu et al. [108] conducted a meta-analysis for CTCs and ctDNA in lung cancer and also observed that the diagnostic performance depends on the detection methods. As described in this review, cfDNA, cfHPV DNA, cfRNAs, CTCs, and EVs in HNSCC investigations were tested in different body fluids (blood and/or saliva) and were also assessed by different techniques for screening a broad of candidate biomarkers, thus providing variability in sensitivity and specificity. Also, HNSCCs involve different anatomic sites and clinical categories, which may result in variable findings depending on the evaluation criteria. Nevertheless, all the studies described in this review are essential and attractive as initial steps to discuss the implementation of liquid biopsy in HNSCCs routine in order to complement the imaging-based assessment. As an emerging area in cancer investigations, further studies are needed to optimize the clinical application of liquid biopsy in HNSCCs to improve diagnosis, prognosis, and treatment planning. The HNSCCs studies highlighted in this review using liquid biopsy and their methodologies are summarized in Table 1.

**Table 1.** Description of liquid biopsy applied in HNC studies highlighted in this review

**Circulating biomarkers**	**Biofluid types**	**HNC category**	**Molecular assays**	**Detection/isolation methods**	**References**
*cfDNA*	Plasma	HNSCC	CNAs	NGS	[11, 12]
		HNSCC	Somatic mutations	ddPCR	[14]
		OPSCC	Somatic mutations	NGS	[16]
		HNSCC	Quantification	Qubit fluorescence	[23]
		OPC	DNA methylation	Q-MSP	[35]
		HNSCC	DNA methylation	qPCR; quasi-digital PCR	[36, 37]
		OPSCC	DNA methylation	ddPCR	[38]
	Plasma and saliva	OSCC	Somatic mutations	NGS	[21]
*cfmtDNA*	Plasma	HNSCC	Quantification	qPCR	[29]
*cfHPVDNA*	Plasma	OPSCC	Quantification	ddPCR	[39, 41, 42]
				qPCR	[40]
*miRNAs*	Plasma	Tongue SCC	Expression	PCR arrays	[49]
		LSCC	Expression	ddPCR; qRT-PCR	[50, 55]
		OSCC	Expression	NGS	[51]
		HNSCC	Expression	qRT-PCR	[54, 57]
		HNSCC	Expression	NGS	[59]
	Serum	OSCC	Expression	qRT-PCR	[52]
		LSCC	Expression	PCR arrays	[53]
		HNSCC	Expression	qRT-PCR	[56]
		LSCC	Expression	qRT-PCR	[58]
	Saliva	OSCC	Expression	qRT-PCR; PCR arrays	[60, 61]
		HNSCC	Expression	qRT-PCR; PCR arrays	[62]
				qRT-PCR; NGS	[63]
				PCR arrays	[64]
	Serum and saliva	Salivary gland tumors	Expression	qRT-PCR	[65]
*lncRNAs*	Plasma	HNSCC	Expression	PCR arrays; NGS	[66]
		OSCC	Expression	qRT-PCR	[67]
		HNC	Expression	Semiquantitative PCR	[68]
		HNSCC	Expression	qRT-PCR	[69]
CTCs	BM and bloodstream	HNSCC	Detection and counting	Immunocytochemistry and fluorescence microscopy	[72, 81]
			Detection	RT-PCR	[73, 79]
	Bloodstream	HNSCC and OSCC	Detection and counting	Ficoll-Hypaque, dextran sedimentation and immunocytochemistry	[74, 76–78]
		HNSCC	Detection and counting	CellSearch^®^	[75, 82]
		LAOC	Detection and counting	RosetteSep^TM^ technique and immunocytochemistry	[83]
		rHNSCC	Detection and counting	EPISPOT, FCM and CellSearch^®^	[84]
		HNSCC	Detection and counting	SERS	[85]
EVs	Plasma	HNSCC	Quantification; characterization	Size-exclusion chromatography; FCM	[93, 94, 96, 99]
		OSCC	Cargo analysis (miRNAs profile)	Commercial kit; qRT-PCR	[100]
		HNSCC	Cargo analysis (miRNAs profile)	Commercial kit; qRT-PCR	[101]
		Tongue SCC	Cargo analysis (protein profile)	Centrifugation and size-exclusion chromatography; isobaric labelling (iTRAQ) and two-dimensional high-performance liquid chromatography followed by tandem mass spectrometry	[104]
	Saliva	OSCC	Cargo analysis (miRNAs profile)	Centrifugation; qRT-PCR array	[102]
				Commercial kit; qRT-PCR array	[103]
			Cargo analysis (protein profile)	Ultracentrifugation; SWATH_MS (Sequential Window Acquisition of all Theoretical Mass Spectra)	[105]

## Conclusions

Liquid biopsy is a promising area of cancer investigation. Despite the challenges in the implementation of liquid biopsy in clinical routine, relevant findings are described in HNSCC studies. Additionally, the screening of circulating HPV DNA in both blood and saliva samples could be useful to describe the disease status in HPV-associated HNSCC. However, few studies in the literature describe the detection of circulating HPV DNA and therefore new studies are potentially welcome for clinical surveillance since the overall HPV prevalence in HNSCC is nearly 26%. The better characterization of candidates for circulating biomarkers and the studies involving a larger number of patients should be considered in liquid biopsies from HNSCC to improve diagnosis and prognosis investigations in order to benefit patients.

## Abbreviations

AUC: area under the curve
BM: bone marrow
cfDNA: cell-free DNA
cfnDNA: cell-free nuclear DNA
cfRNA: cell-free RNA
CKs: cytokeratins
CNAs: copy number aberrations
CNI: copy number instability
CRP: C-reactive protein
CTCs: circulating tumor cells
ctDNA: circulating tumor DNA
ddPCR: droplet digital polymerase chain reaction
DFS: disease-free survival
DMFS: distant metastasis-free survival
EPCAM: epithelial cell adhesion molecule
EVs: extracellular vesicles
FCM: flow cytometry
HNC: head and neck cancer
HNSCC: head and neck squamous cell carcinoma
HPV: human papillomavirus
IHC: immunohistochemistry
lncRNAs: long non-coding RNAs
LSCC: laryngeal squamous cell carcinoma
miRNA: microRNA
mtDNA: mitochondrial DNA
NGS: next-generation sequencing
OPC: oropharyngeal cancer
OPSCC: oropharyngeal squamous cell carcinoma
OS: overall survival
OSCC: oral squamous cell carcinoma
p16: cyclin-dependent kinase inhibitor 2A
PD-L1: programmed death-ligand 1
PFS: progression-free survival
qPCR: quantitative/real-time polymerase chain reaction
qRT-PCR: quantitative reverse transcription polymerase chain reaction
TNM: tumor, lymph nodes, metastasis

## Declarations

### Copyright

© The Author(s) 2022.
